# MicroRNA-1269 promotes proliferation in human hepatocellular carcinoma via downregulation of FOXO1

**DOI:** 10.1186/1471-2407-14-909

**Published:** 2014-12-03

**Authors:** Xue-Wei Yang, Guan-Zhu Shen, Liang-Qi Cao, Xiao-Feng Jiang, He-Ping Peng, Gang Shen, De Chen, Ping Xue

**Affiliations:** Department of Hepatobiliary Surgery, the Second Affiliated Hospital of Guangzhou, Medical University, Guangzhou, 510260 China; Department of Radiation Oncology, Sun Yat-Sun University Cancer Center; State Key Laboratory of Oncology in South China, Collaborative Innovation Center for Cancer Medicine, Guangzhou, 510318 China; Department of Interventional Therapy and Vascular Anomalies, Guangzhou Women and Children’s Medical Center, Guangzhou, 510623 China

**Keywords:** miR-1269, Hepatocellular cancer, Proliferation, FOXO1

## Abstract

**Background:**

Hepatocellular carcinoma (HCC) is one of the most common malignancies and a major cause of cancer-related mortality in the world. MicroRNAs (miRNAs) are small, noncoding RNAs that play essential roles in various stages during cancer progression. The aim of the current study was to elucidate the role of miR-1269 in the pathogenesis of HCC.

**Methods:**

The expression of miR-1269 in HCC cells and tissues were determined by Real-time PCR analysis. Cell viability, colony formation and anchorage-independent growth ability assays were performed to examine cell proliferative capacity and tumorigenicity. Flow cytometry analysis was conducted to determine cell cycle progression. The expression of p21, CyclinD1, phosphorylated Rb, Rb and FOXO1 were examined by Western blotting analysis. Luciferase assay was used to determine whether FOXO1 is the direct target of miR-1269.

**Results:**

miR-1269 was upregulated in HCC cells and tissues. Ectopic miR-1269 expression promoted, but inhibition of miR-1269 reduced, proliferation, tumorigenicity and cell cycle progression of HCC cells. Furthermore, we demonstrated that FOXO1 was a direct target of miR-1269. Suppression of FOXO1 by miR-1269 was associated with dysregulation of p21, cyclin D1, phosphorylated Rb and Ki67 expression, thereby playing an essential role in the growth of HCC cells.

**Conclusions:**

Our study indicated that overexpression of miR-1269 promotes cell proliferation in HCC through directly suppressing FOXO1, and functions as an oncomiR in HCC.

## Background

Hepatocellular carcinoma (HCC) is one of the most common cancers in the world, and the third most common cause of cancer-related death, especially in Asian countries [1, 2]. HCC is particularly problematic in China, where the incidence of HCC is much higher than that in other Asian countries [2]. Most patients with HCC are asymptomatic until the later stages of disease, and the lack of an effective way of diagnosis at an earlier stage means the these patients have a poor prognosis [3]. The identification of specific and effective diagnostic biomarkers for HCC is of critical importance. Therefore, studies into the biology of HCC initiation and progression are also urgently needed to develop effective therapeutic strategies.

MicroRNAs (miRNAs) are a class of newly recognized small noncoding RNAs, which have been demonstrated to be as important factors in multiple biological processes [4–7]. It has been shown that miRNAs negatively regulate gene expression levels post-transcriptionally through targeting the 3’-untranslated region (3’-UTR) of mRNAs in a sequence-specific manner [8, 9]. An increasing number of studies have shown that miRNAs play essential roles in the biology of various human cancers through regulation of multiple biological process, such as cell growth and differentiation, apoptosis, metastasis and angiogenesis [6, 10, 11]. Based on the critical role of miRNAs in carcinogenesis and cancer progression, miRNAs have been considered as putative targets for cancer diagnosis and therapy.

The regulation of miRNAs during HCC progression has been arousing increasing attentions recently. Alexander H. *et al.* reported that the pan-deacetylase inhibitor panobinostat suppresses the expression of oncogenic miRNAs in HCC cell lines and anobinostat exerts its anti-cancer effect by suppressing these miRNAs and restoring the expression of their corresponding tumor suppressor targets [12]. Panobinostat strongly downregulated High Mobility Group AT-2 hook (HMGA2), a nuclear non-histone transcriptional co-factor with known oncogenic properties, in HepG2 and Hep3B cells and the effect was found to be mediated by transcriptional upregulation and promotion of the maturation of the tumor suppressor miRNA hsa-let-7b, which could inhibit HMGA2 expression via RNA interference pathways [13]. However, the network regulation of miRNAs in HCC progression has not been elucidated clearly.

In the current study, we found that miR-1269 was upregulated through analysis of a published micro-array-based high-throughput assessment (NCBI/GEO/GSE36915), and further confirmed this result in HCC tissue and cell lines. Ectopic overexpression of miR-1269 in HCC cell lines led to the promotion of cell growth rate, tumorigenicity and cell cycle progression. Furthermore, we demonstrated that the tumor suppressor gene FOXO1 is a direct target of miR-1269. In conclusion, our results indicated that overexpression of miR-1269 could promote cell proliferation, tumorigenicity and cell cycle progression in HCC by directly suppressing FOXO1.

## Methods

### Cell culture

Immortalized normal liver epithelial cell, THLE3, was purchased from the American Type Culture Collection (ATCC, Manassas, VA, USA). The HCC cell lines (Hep3B, HepG2, BEL-7402, BEL-7404, SNU-398, SNU-449, Huh7, and QGY-7703), were purchased from the ATCC, were maintained in Dulbecco’s modified Eagle’s medium (Invitrogen, Carlsbad, CA) supplemented with 10% fetal bovine serum (Invitrogen), 100 U/ml penicillin and 100 μg/ml streptomycin (Invitrogen), within a humidified atmosphere containing 5% CO_2_ at 37°C. Normal hepatocytes s established from fresh specimens of normal hepatic tissue, which had been histopathologically diagnosed and verified by experienced pathologists.

### Tissue specimens

A total 23 pairs of HCC tumors and matched normal tissue from adjacent regions, which were diagnosed histopathologically by experienced pathologists, were used in this study. Fresh HCC tissues and normal hepatic tissues were collected from patients undergoing curative resection and diagnosed histopathologically at the department of hepatobiliary surgery in the Second Affiliated Hospital of Guangzhou Medical University. All samples were immediately frozen and stored in liquid nitrogen before further analysis. All samples were obtained with informed consent and this study was approved by of Sun Yat-sen University Cancer Center Institutional Review Board.

### Plasmid, siRNA and generation of stably engineered cell lines

The miR-1269 expression plasmid was generated by cloning the genomic pre-miR-1269 gene into the retroviral transfer plasmid pMSCV-puro (Clontech Laboratories, Mountain View, CA, USA). The miR-1269 mimic, miR-1269 mutant mimic (miR-1269-mut), miR-1269 inhibitor , negative control (NC) and FOXO1 siRNA were purchased from RiboBio (RiboBio, Guangzhou, Guangdong, China). Transfection of oligonucleotides and siRNA were performed using Lipofectamine 2000 reagent (Invitrogen, Carlsbad, CA, USA), according to the manufacturer’s instructions. The stably engineered pMSCV-miR-1269 cell line was established using standard methods [14]. Briefly, pMSCV-miR-1269 was cotransfected with the packaging plasmid into 293FT cells. Cell supernatants were then collected after 36 h and incubated with HCC cell lines for 24 h in the presence of polybrene (2.5 μg/ml; Sigma, Saint Louis, MO, USA). Puromycin (1.5 μg/ml; Sigma) was used to select stably transduced cells over a 10 day time period, according to the manufacturer’s instructions.

### RNA isolation and quantitative reverse-transcription PCR (qRT-PCR)

Total cellular RNA was extracted using Trizol reagent (Invitrogen), according to the manufacturer’s protocol. SYBR Green I (Molecular Probes, Invitrogen) was used to quantify PCR amplification and the qRT-PCR was performed and analyzed using a 7500 Fast Real-Time Sequence detection system (Applied Biosystems, Foster City, CA, USA). miRNA quantification was determined by using Bulge-loop™ miRNA qRT-PCR Primer Set (one RT primer and a pair of qPCR primers for each set) specific for miR-1269, designed by RiboBio. The relative expression levels of the miRNA were calculated as 2^-[(Ct of miRNA) – (Ct of U6)]^ after normalization with reference to the expression of small nuclear RNA U6. The primers used for stem-loop reverse-transcription PCR for miR-1269 and U6 were purchased from RiboBio. Expression levels of genes were normalized to that of the housekeeping gene *GAPDH* as the control and calculated as 2^-[(Ct^^of *GENES*) – (Ct^^of *GAPDH*)]^). The following primers were used: p21 forward, 5’-CATGGGTTCTGACGGACAT-3’, p21 reverse, 5’-AGTCAGTTCCTTGTGGAG CC-3’; p27 forward, 5’-TGCAACCGACGATTCTTCTACTCA A-3’, Cyclin D1 forward, 5’-AACTACCTGGACCGCTTCCT-3’, and Cyclin D1 reverse, 5’-CCACTTGAGCTTGTT CACCA-3’.

### Western blotting

Total protein was extracted from whole cells and 20 μg of isolated protein was separated by SDS-PAGE and electroblotted onto a PVDF membrane (Bio-Rad Laboratories, Hercules, CA, USA). The membranes were then probed with antibodies against FOXO1, p21, CyclinD1, phosphorylated Rb, Rb, and α-Tubulin (Cell Signaling, Danvers, MA, USA), using standard protocols.

### Cell viability assay

Cells were seeded onto 96-well plates (2 × 10^3^ cells per well), and 100 μl 3-(4, 5-Dimethyl-2-thiazolyl)-2, 5-diphenyl-2H-tetrazolium bromide (MTT, 0.5 mg/ml, Sigma) was added at the indicated time points and incubated for 4 h at 37°C. DMSO (150 μl) was then added after removal of the culture medium (Sigma), and the absorbance was measured at 570 nm, with 655 nm as the reference wavelength.

### Colony formation assay

Cells were seeded into 6-cm tissue culture dishes (0.5 × 10^3^ cells per well) and cultured for 14 days. Cells were then fixed with 10% formaldehyde for 15 min and subsequently stained with 1.0% crystal violet for 5 min. The number of colonies formed was counted in 10 different fields.

### Anchorage-independent growth ability assay

Cells (1× 10^3^) were trypsinized and suspended in 2 ml of complete medium plus 0.33% agar (Invitrogen), and plated in 6-cm culture dishes on top of a bottom agar layer (0.66% complete medium agar), and incubated at 37°C for 2 weeks. Colonies greater than 0.1 mm in diameter were counted.

### Flow cytometry

Cells were harvested by trypsinization, washed in ice-cold PBS, and fixed in 75% ice-cold ethanol. Cells were then treated with Bovine pancreatic RNAase (2 μg/ml; Sigma) at 37°C for 30 min, followed by incubation with propidium iodide (20 μg/ml; Sigma) for 20 min. Cell cycle analysis was determined using a BD LSRII Flow Cytometry System with FACSDiva software (BD Bioscience, Franklin Lakes, USA).

### Luciferase assay

A region of the human FOXO1 3’-UTR containing three miR-1269 binding sites was amplified by PCR and cloned into vector pGL3 (Promega, Madison, WI, USA). Cells were seeded in triplicate in 24-well plate and allowed to settle for 24 h. One hundred nanograms of pGL3-FOXO1-luciferase plasmid were transfected into HCC cells using the Lipofectamine 2000 reagent. Luciferase and control signals were measured at 48 h after transfection using the Dual Luciferase Reporter Assay Kit (Promega), according to the protocol provided by the manufacturer.

### Statistical analysis

Student’s t-test was used to evaluate the significant difference between two groups of data in all the pertinent experiments. All data were expressed as the mean ± standard deviation (SD) for three independent experiments. Results were considered statistically significant when a P-value < 0.05 was obtained.

## Results

### MiR-1269 is upregulated in HCC tissues and cells

**Figure 1:**
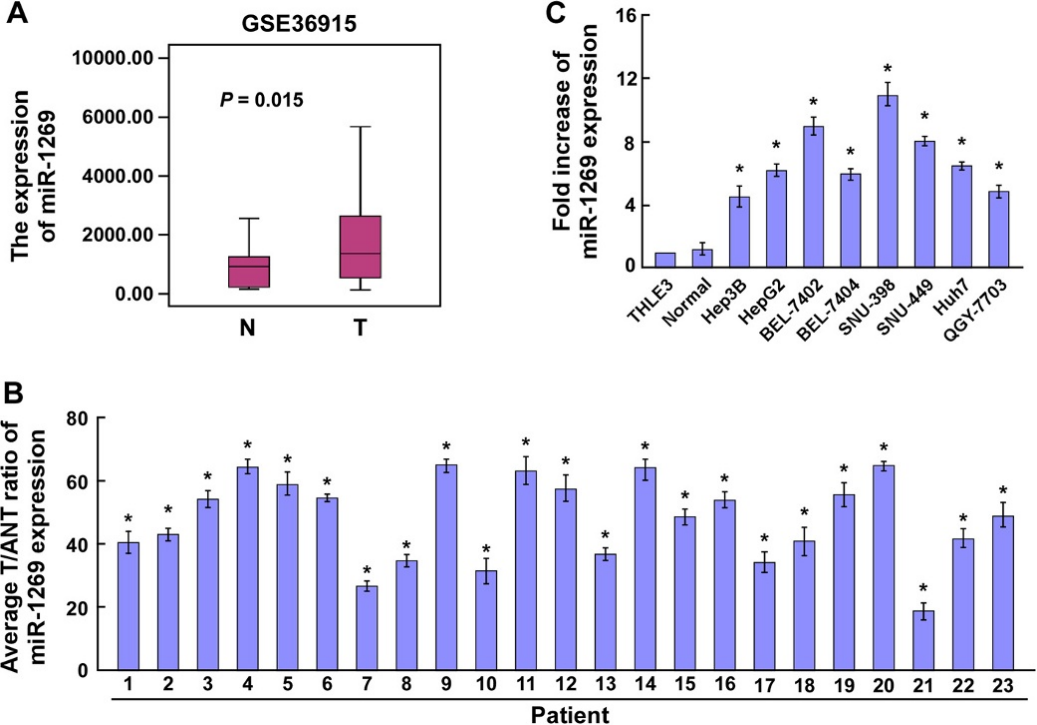
**MiR-1269 is upregulated in HCC. A**. miR-1269 is upregulated in HCC tissues compared with normal liver tissue (*P* = 0.015; NCBI/GEO/ GSE36915). **B**. Real-time PCR analysis of miR-1269 expression in 23 paired cancerous tissues (T) and their adjacent noncancerous hepatic tissues (ANT). Transcript levels were normalized to U6 expression. **C**. Real-time PCR analysis of miR-1269 expression in several HCC cell lines compared to normal hepatic cells as control. Transcript levels were normalized to U6 expression. Each bar represents the mean ± SD of three independent experiments. * *P* < 0.05.

By analyzing a published micro-array-based high-throughput assessment (NCBI/GEO/GSE36915; n (Non-tumor) = 21; n (Tumor) =68), miR-1269 was found to be significantly upregulated in HCC tissues, compared with that in non-tumor tissues (*P* < 0.05; Figure 1A). We further examined the expression level of miR-1269 in HCC tissues and cell lines by qRT-PCR. As shown in Figure 1B, miR-1269 was upregulated in all 23 pairs of HCC tissue, compared with the adjacent noncancerous hepatic tissues. Moreover, we found that miR-1269 was also upregulated in eight HCC cell lines (Hep3B, HepG2, BEL-7402, BEL-7404, MHCC97H, MHCC97L, Huh7, and QGY-7703), compared with that in the normal liver epithelial cell line THLE3 cells and normal hepatocytes (Normal) (Figure 1C). Taken together, these data demonstrate that miR-1269 expression is elevated in HCC tissues and cell lines.

### Ectopic overexpression of miR-1269 promotes proliferation of HCC cells

**Figure 2:**
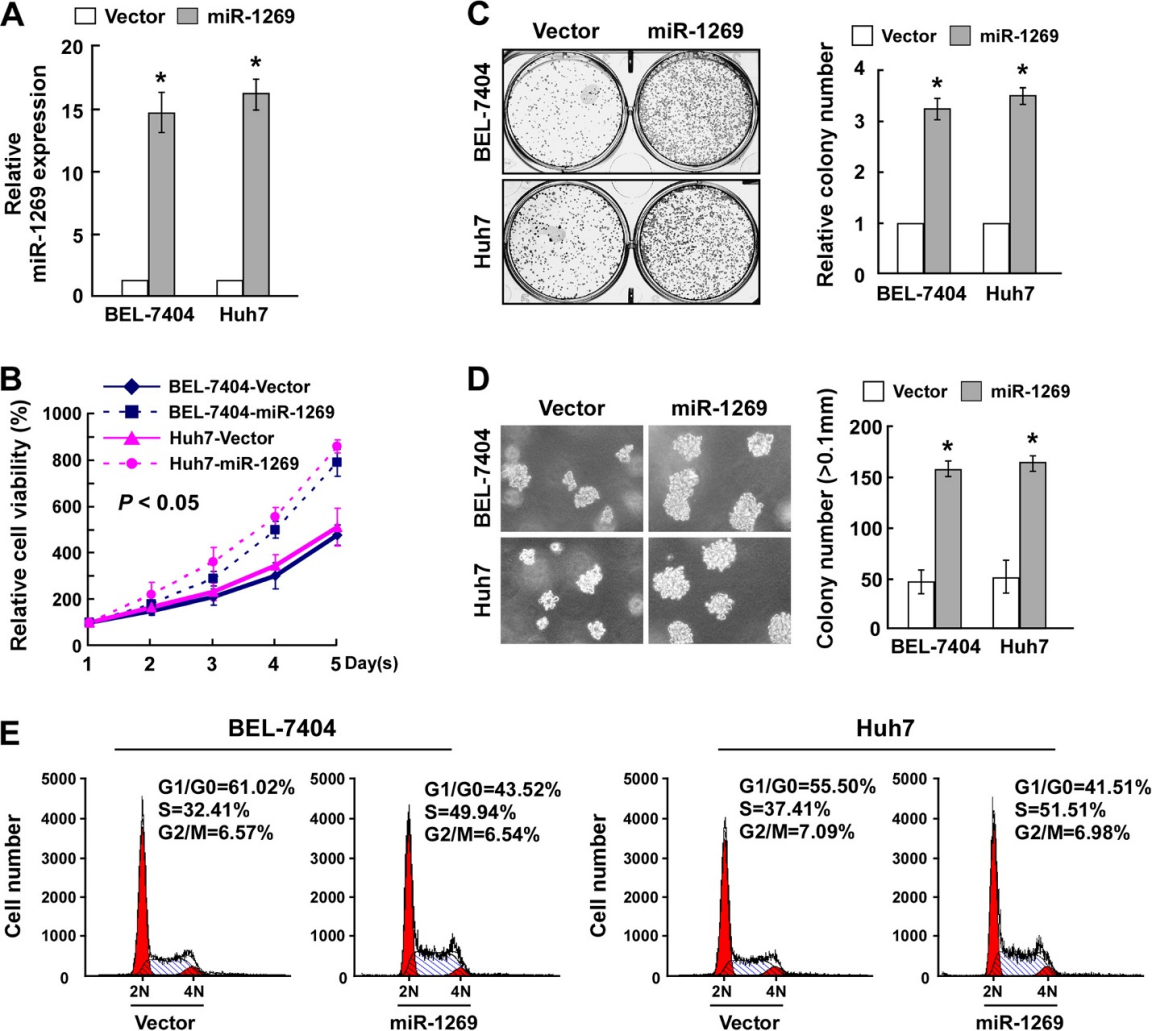
**MiR-1269 enhances the proliferation and tumorigenicity of HCC cells. A**. Real-time PCR analysis of miR-1269 in BEL-7404 and Huh7 cells stably overexpressing miR-1269. Transcript levels were normalized to U6 expression. **B**. The effects of miR-1269 overexpression on cell viability of the indicated cell lines analyzed by MTT assay. **C**. Representative micrographs (left panel) and quantification (right panel) of crystal violet stained colonies formed by the indicated cell lines. **D**. Representative micrographs (left panel) and quantification (right panel) of cell colonies determined by anchorage-independent growth ability assay. **E**. The effect of miR-1269 on cell cycle progression of the indicated cell lines analyzed by flow cytometry. Each bar represents the mean ± SD of three independent experiments. * *P* < 0.05.

To explore the biological function of miR-1269 in HCC progression, BEL-7404 and Huh7 cells stably overexpressing miR-1269 were established (Figure 2A). Cell viability was then measured by MTT assay. A shown in Figure 2B, ectopic expression of miR-1269 increased the growth rate of both HCC cell lines. Colony formation assay showed that upregulation of miR-1269 promoted the colony formation capacity of BEL-7404 and Huh7 cells (Figure 2C). Consistently, anchorage-independent growth assay revealed that the cells stably expressing miR-1269 formed more and larger-sized colonies than the control cells (Figure 2D). Furthermore, cell cycle analysis by flow cytometry showed a dramatic decrease in the percentage of cells in the G1/G0 phase and an increase in the percentage of cells in the S phase in miR-1269-overexpressing cells (Figure 2E). Collectively, these results suggest that upregulation of miR-1269 enhanced the proliferation, tumorigenicity and cell cycle progression of HCC cells.

### Inhibition of miR-1269 suppresses proliferation of HCC cells

**Figure 3:**
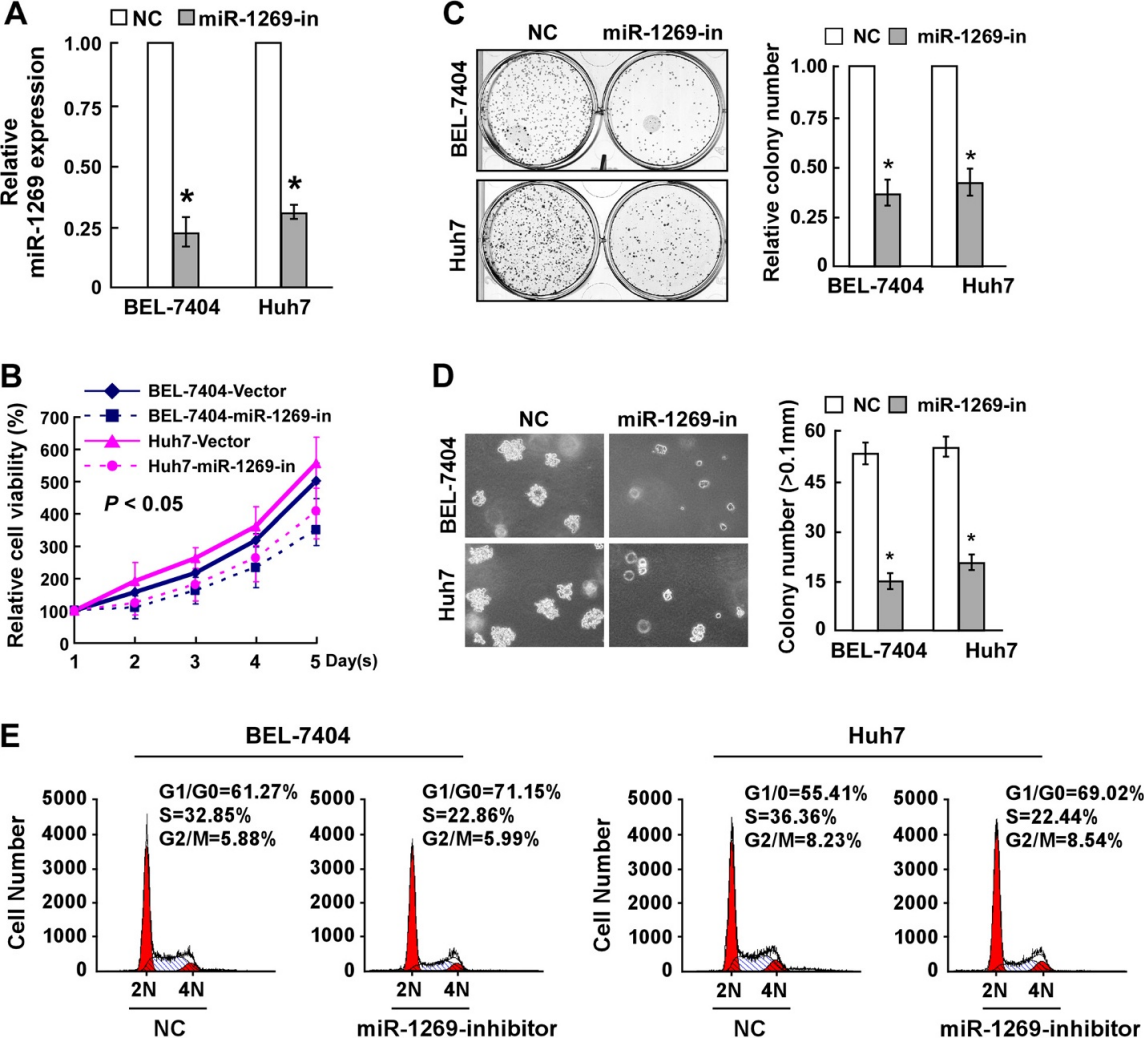
**Inhibition of miR-1269 suppresses the proliferation and tumorigenicity of HCC cells. A**. Real-time PCR analysis miR-1269 in BEL-7404 and Huh7 cells transfected with a miR-1269 inhibitor. Transcript levels were normalized to U6 expression. **B**. The effects of miR-1269 inhibition on cell viability of the indicated cell lines analyzed by MTT assay. **C**. Representative micrographs (left panel) and quantification (right panel) of crystal violet stained colonies formed by the indicated cell lines. **D**. Effects of miR-1269 inhibition on the tumorigenicity of the indicated cell lines determined by anchorage-independent growth ability assay. **E**. The effect of miR-1269 on cell cycle progression of the indicated cell lines analyzed by flow cytometry. Each bar represents the mean ± SD of three independent experiments. * *P* < 0.05.

To further explore the role of miR-1269 in promoting HCC cell proliferation, loss of function approach using a miR-1269 inhibitor were performed (Figure 3A). Analysis by MTT assay showed that downregulation of miR-1269 markedly decreased the growth rate of BEL-7404 and Huh7 HCC cells transfected with the miR-1269 inhibitor, compared with that of control cells (Figure 3B). The colony formation and anchorage-independent growth assays both revealed that HCC cells transfected with the miR-1269 inhibitor produced fewer and smaller colonies than the NC cells (Figure 3C and D). Moreover, the result of flow cytometry showed an obvious increase in the percentage of cells in the G1/G0 phase and a decrease in the percentage of cells in the S phase in miR-1269 inhibited cells, compared with control cells (Figure 3E). Taken together, these results suggest that inhibition of miR-1269 suppresses the proliferation, tumorigenicity and cell cycle progression of HCC cells.

### FOXO1 is a direct target of miR-1269 in HCC cells

**Figure 4:**
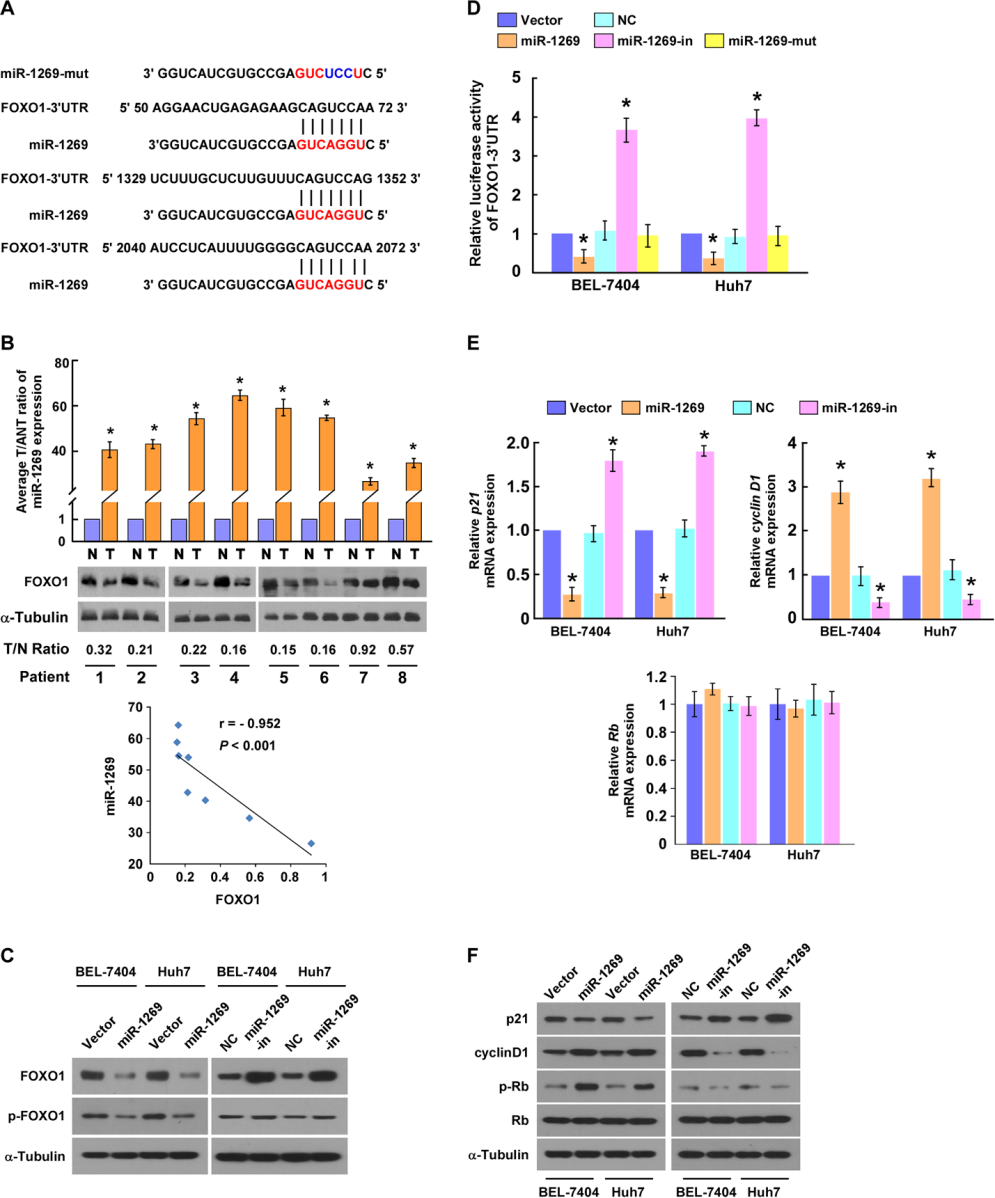
**FOXO1 is a direct target of miR-1269. A**. Sequence alignment of miR-1269, miR-1269 mutant (miR-1269-mut), and putative *FOXO1*-3’UTR. **B**. Real-time PCR analysis of miR-1269 and western blotting analysis of FOXO1 in 8 paired cancerous tissues (T) and their adjacent noncancerous hepatic tissues (N). The quantification of western blotting bands is performed by OD value determined by Quantity One 4.6.2 software. **C**. Western blotting analysis of expression levels of FOXO1 and phosphorylated FOXO1 in the indicated cells. α-Tubulin served as the loading control. **D**. The FOXO1 luciferase reporter activity in the indicated cells transfected with miR-1269 mimic, or miR-1269-mut, miR-1269 inhibitor, or negative control. **E**. Real-time PCR analysis of mRNA expression of *p*21, *Cyclin D1* and *Rb* in the indicated HCC cell lines. **F**. Western blotting analysis of p21, cyclin D1, Ki67, p-Rb, and Rb in the indicated HCC cells. α-Tubulin served as the loading control. Each bar represents the mean ± SD of three independent experiments. * *P* < 0.05.

It is known that miRNAs function by negatively regulating mRNAs via targeting their 3’UTRs. Thus, we used publicly available algorithms (TargetScan 6.2) to predict the potential targets of miR-1269. As shown in Figure 4A, there are three miR-1269 binding sites in the *FOXO1* mRNA 3’UTR, including one conserved and two poorly conserved binding sites. Importantly, western blotting analysis showed that FOXO1 expression was downregulated in the eight pairs of HCC tissue, compared with the adjacent noncancerous hepatic tissues, and statistical analysis revealed that miR-1269 levels inversely correlated with the expression of FOXO1 in the clinical HCC samples (Figure 4B), suggesting that miR-1269 might play role in regulation of FOXO1 in HCC. Indeed, FOXO1 expression was found to be downregulated in the miR-1269-overexpressing BEL-7404 and Huh7 cells but upregulated in the cells transfected with miR-1269 inhibitor. In addition, overexpressing miR-1269 decreased the expression of p-FOXO1, but we did not found significant alterations of FOXO1 in miR-1269-inhibited cells (Figure 4C). To further confirm the direct regulation of FOXO1 by miR-1269, the pGL3-*FOXO1*-3’-UTR-luciferase reporter, containing the three putative miR-1269 binding sites, was constructed. The luciferase assay results showed that ectopic overexpression of miR-1269 decreased, but inhibition of miR-1269, increased luciferase activity of the pGL3-*FOXO1*-3’-UTR reporter (Figure 4D). Meanwhile, mutant miR-1269 failed to show an inhibitory effect on luciferase expression driven by the pGL3-*FOXO1* -3’-UTR-luciferase reporter (Figure 4D). Collectively, these results suggest that miR-1269 directly targets FOXO1 in HCC cells.

Given that our results indicated miR-1269 could influence HCC cell proliferation, we investigated its effects on the expression level of FOXO1 downstream genes, such as p21^Cip1^, cyclin D1, and Rb. As shown in Figure 4E and F, p21^Cip1^ was downregulated, but cyclin D1 and phosphorylated Rb, were upregulated in miR-1269-transfected cells. As expected, p21^Cip1^ was upregulated, whereas cyclin D1 and phosphorylated Rb were downregulated, in miR-1269-inhibitor transfected cells (Figure 4E and F). These results suggest that miR-1269 plays an important role in the proliferation of HCC cells via regulation of FOXO1.

### Suppression of FOXO1 is essential for miR-1269-induced cell proliferation in HCC

**Figure 5:**
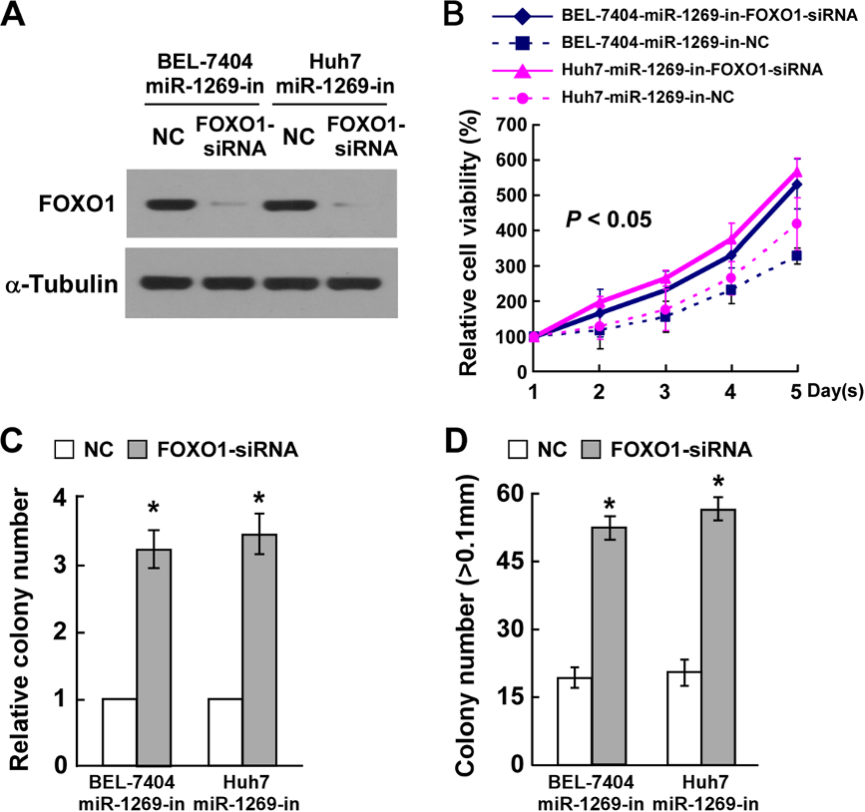
**MiR-1269 promotes proliferation of HCC cells by suppressing FOXO1. A**. Western blotting analysis of FOXO1 in the indicated cells transfected with FOXO1-siRNA. α-Tubulin served as the loading control. **B**. The effects of FOXO1 on miR-1269 mediated HCC proliferation analyzed by MTT assay. **C**. The quantification of crystal violet stained colonies formed by the indicated cells transfected with FOXO1-siRNA or NC. **D**. Quantification of colony numbers of indicated cells treated with FOXO1-siRNA or NC, which determined by anchorage-independent growth assay. Error bars represent the mean ± SD from three independent experiments. * *P* < 0.05.

To further confirm the effect of FOXO1 suppression on miR-1269-mediated proliferation of HCC cells, siRNA assay was used to suppress endogenous FOXO1 expression (Figure 5A). Analysis by MTT assay indicated that suppression of FOXO1 in cells transfected with the miR-1269 inhibitor increased the growth rate of HCC cells (Figure 5B). The colony formation and anchorage-independent growth assays both showed that silencing FOXO1 expression could reverse the inhibitory effect of the miR-1269 inhibitor on HCC cell proliferation (Figure 5C and D). These results further confirm that miR-1269 enhances HCC cell proliferation and tumorigenicity by downregulation of FOXO1 expression, and that FOXO1 suppression is essential for miR-1269-mediated effects on HCC cell proliferation and tumorigenicity.

## Discussion

The key finding of the current study is that miR-1296 is significantly upregulated in HCC cells and tissues, compared with normal hepatocytes and liver tissues. Furthermore, we found that ectopic overexpression of miR-1296 promoted the proliferation, tumorigenicity and cell cycle progression of HCC cells. In agreement with these observations, upregulation of miR-1296 decreased the expression of p21^Cip1^, a cyclin-dependent kinase (CDK) inhibitor, and increased the expression of the cell cycle regulator cyclin D1 and Ki67. Moreover, we demonstrated that miR-1296 suppressed FOXO1expression via directly targeting its 3’-UTR. Taken together, our results suggest that upregulation of miR-1296 might play an important role in promoting carcinogenesis and progression of HCC.

FOXO proteins are a subfamily of the forkhead transcription factors [15]. FOXO1 is a potent transcriptional activator that triggers the expression of a program of genes involved in cell cycle arrest, apoptosis, DNA repair and hypoxia responsiveness [16–19]. FOXO1 is also known as a tumor suppressor and its deregulation has been identified in various tumors [20]. Typically, inhibition of FOXO1 in cancer is associated with the activation of higher level kinases such as Akt and IKK (IκB kinase) [15, 17]. It is known that activated Akt leads to FOXO1 phosphorylation, resulting in FOXO1 translocating from the nucleus to cytoplasm by binding with the 14-3-3 chaperone protein, and then subsequently undergoing protein degradation [15, 17]. The regulation of FOXO1 by miRNAs has been reported elsewhere. For example, Anna *et al*. found that FOXO1 is a bona fide target of miR-182 and mediated the function of miR-182 in promoting clonal expansion of activated helper T lymphocytes [21]. Furthermore, three microRNAs, miR-27a, miR-96, and miR-182, have all been found to directly target FOXO1 and regulate endogenous FOXO1 protein expression in breast cancer cells, while suppression of these microRNAs resulted in an increase in FOXO1 protein and a decrease in cell growth [22]. It is also known that mir-223 regulates cell proliferation through targeting and downregulating FOXO1 [23], and upregulation of miR-370 promotes proliferation of prostate cancer cells by suppressing the expression FOXO1 [24]. In the present study, we found that FOXO1 is a direct target of miR-1269, which could downregulate its expression. These results supported the viewpoint that, in addition to the regulation of FOXO1 by phosphorylation, there are other regulatory mechanisms that can modulate FOXO1 expression, i.e. epigenetic regulation by miRNAs. Moreover, consistent with previous studies, the regulation of FOXO1 by specific miRNAs is essential in tumor development and progression.

CDK1 and CDK2, two cell cycle regulatory protein kinases that play important roles in cell cycle transitions, have been reported to phosphorylate FOXO1 and attenuate its tumor suppressor function [25]. It has also been reported that upregulation of the constitutively active nuclear form of FOXO1 represses the activity of CDK4, which is important for G1 cell cycle progression controlled by cyclin D [26]. Moreover, previous studies have shown that FOXO subfamily members play critical roles in suppressing tumor growth by increasing the expression level of the cell cycle inhibitor p21^Cip1^ and p27^Kip1^, and decreasing the expression of the cell cycle regulator cyclin D, consequently leading to G1/S cell cycle arrest [24, 27, 28]. Consistent with this literature, we found that ectopic miR-1269 inhibits the expression of p21 ^Cip1^, and induces the expression of cyclin D1.

Dalmasso, *et al*. found that transfection of enterocyte-like Caco2-BBE cells with antisense of mature miR-1269 could decrease growth rate and trans-epithelial resistance of the cells, indicating their shift toward colonocyte-like HT29-Cl cells, which is one clone of human colonic cancer HT29 cells [29].

## Conclusion

In conclusion, this study revealed that miR-1269 might have potential function to determine human intestinal epithelial cell fate. However, the function and regulation of miR-1269 during HCC progression has not been unfolded. Herein, for the first time we have revealed an important link between miR-1269 and HCC progression. We have shown that miR-1269 functions in regulating cell growth, tumorigenicity, and cell cycle progression. This work adds to the growing body of knowledge concerning the essential role that miRNAs play in the pathogenesis of cancer, and further suggests that miR-1269 is an oncomiR and might represent a potential therapeutic target for HCC.
